# Porous Graphene Sponge Additives for Lithium Ion Batteries with Excellent Rate Capability

**DOI:** 10.1038/s41598-017-01025-7

**Published:** 2017-04-19

**Authors:** Qian Cheng

**Affiliations:** IoT Devices Research Laboratories, NEC Corporation, Tsukuba, Ibaraki 305–0817 Japan

## Abstract

Rate capability as well as power performance of lithium ion batteries (LiBs) is becoming more and more important, especially as the application targets of LiBs move from mobile devices to transportation, such as EVs and HEVs. In this research, we report porous graphene sponge additives for both anode and cathode materials for better rate performance. The charge capacity retention improved from 56% to 77% at 6C and from 7% to 45% at 10C with 0.5 wt% added to the anode, while the discharge capacity retention at the 6C rate improved from 43% to 76% and the 10C rate discharge improved from 16% to 40% with the same amount of MG added to the cathode. The cyclability at high rate was also improved with the MG additive. Moreover, preparation of the MG was facile, cost-effective, and compatible with commercially available active materials. These results demonstrate the suitability of MG for use with LiB additives to ensure better rate capability and high rate cyclability.

## Introduction

Over the last two decades, lithium ion batteries have become more highly desired as mobile devices and energy efficient transportation such as hybrid electric vehicles (HEVs) and electric vehicles (EVs) as well as stationary energy storage devices for smart energy management systems continue to evolve^1–6^. The current commercially available lithium ion batteries with a graphite anode and layer-structure LiMO_2_ (M = Mn, Ni, Co binary or ternary system) cathode have a gravimetric energy density more than 160 Wh/kg at the cell level but suffer from low power performance such as poor charge and discharge rate capability and high-rate cyclability^7–11^. This is because energy density focused cell design generally calls for high mass loading on both the anode (>200 g/m^2^ for double side deposition) and cathode (>450 g/m^2^ for double side deposition), low electrolyte coefficient, low porosity of both anode and cathode (<25%), little conductive additive usage (<3 wt%), and active material with low surface area, which result in high energy density LiBs with poor power performance^12, 13^. Currently, the power performance of a LiB, such as charging time and pulse power supply, is becoming more and more important, especially as the application targets move from small mobile devices to electric vehicles, since EV users, for example, are hardly willing to wait more than half an hour to charge their cars during a long drive compared to a less than 5-min gasoline refueling^4, 14–17^. As a result, it is very important to increase the power performance of lithium ion batteries.

Some of the strategies used in the battery industry to increase the power performance of the LiB include 1) lower mass loading of electrodes for better electrolyte accessibility at high rates, 2) larger weight proportion of conductive additives in both anode and cathode for better electrode conductivity, 3) larger electrode porosity for more electrolyte absorption, and 4) the use of alternated electrode materials such as amorphous carbon or lithium titanium oxide (LTO) as anode materials for fast lithiation^18–21^. Although the power performance can be improved by cell design engineering, this leads to low energy density and high $/Wh of the cell.

**Figure 1:**
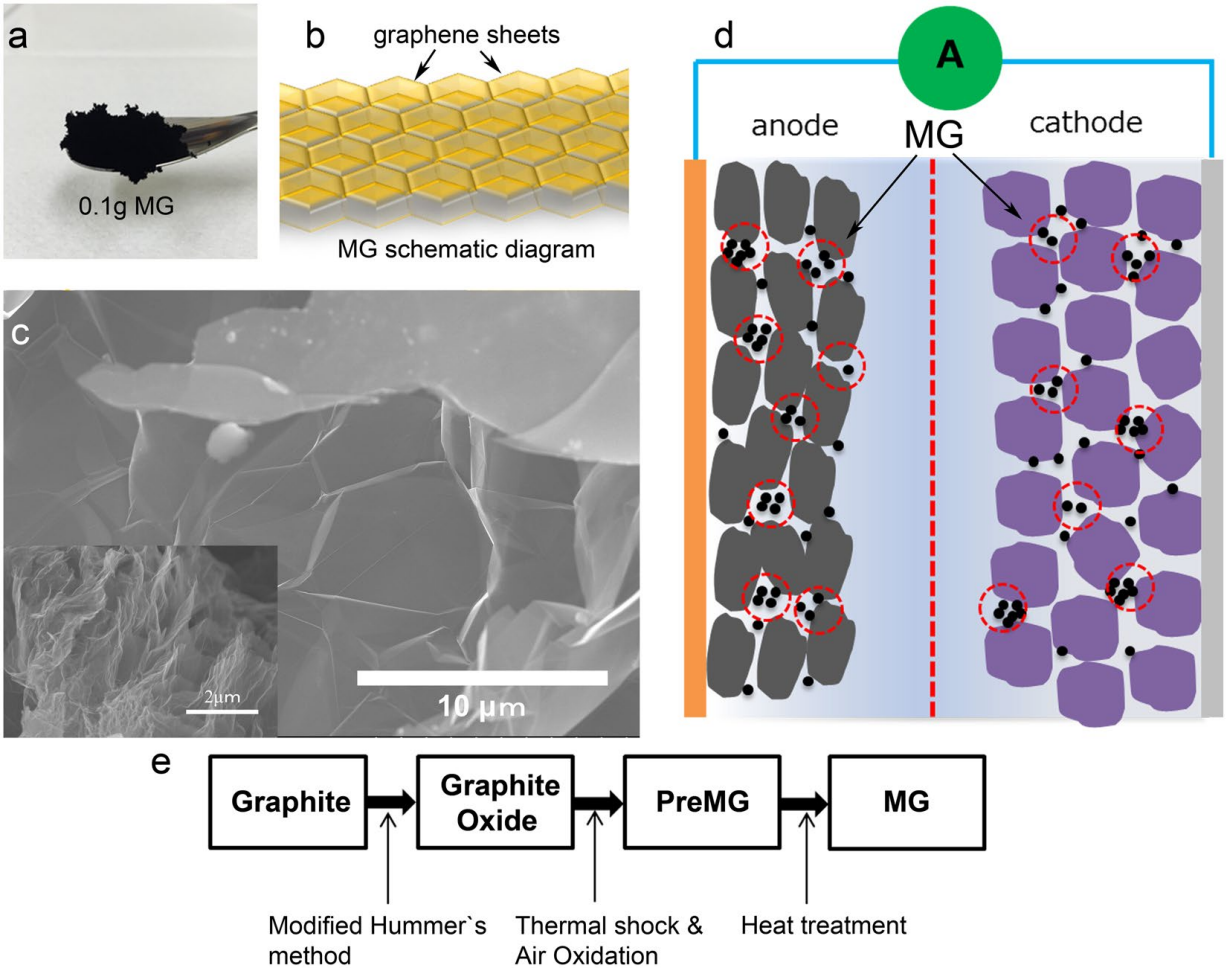
Schematic diagram and the material synthesis process. (**a**) Digital photo of 0.1 g Magic G featuring ultralow density that can even flow in air. (**b**) Schematic diagram of MG with honeycomb-like structure. The wall of each honeycomb cell is made of graphene sheets. (**c**) SEM image of synthesized MG. The honeycomb cell structure graphene sheets can be indicated (MG in low magnification SEM was inserted). (**d**) The MG can be used as an additive for both anode and cathode. Schematic diagram of lithium ion battery consisting of anode, cathode, electrolyte, and separator. (**e**) Synthesis method of Magic G. Natural graphite is used as the raw material and a modified Hummer’s method is used to make graphite into graphite oxide (GO). The GO is then thermal shocked in N_2_ to 400 °C in 10 min and mild oxidized in air for 30 min at 500 °C to form the Magic G precursor (PreMG). Finally, the PreMG is heat treated at 1000 °C for 4 h in N_2_ to obtain MG.

In this study, we have developed a honeycomb-like porous graphene sponge called “Magic G” (MG) with high electric conductivity, high specific surface area, and high ability of electrolyte absorption as an additive for both the anode and cathode materials of LiBs that can increase the rate capability and high rate cyclability of the lithium ion battery. This material is shown schematically in Fig. 1.

## Results and Discussion

**Figure 2:**
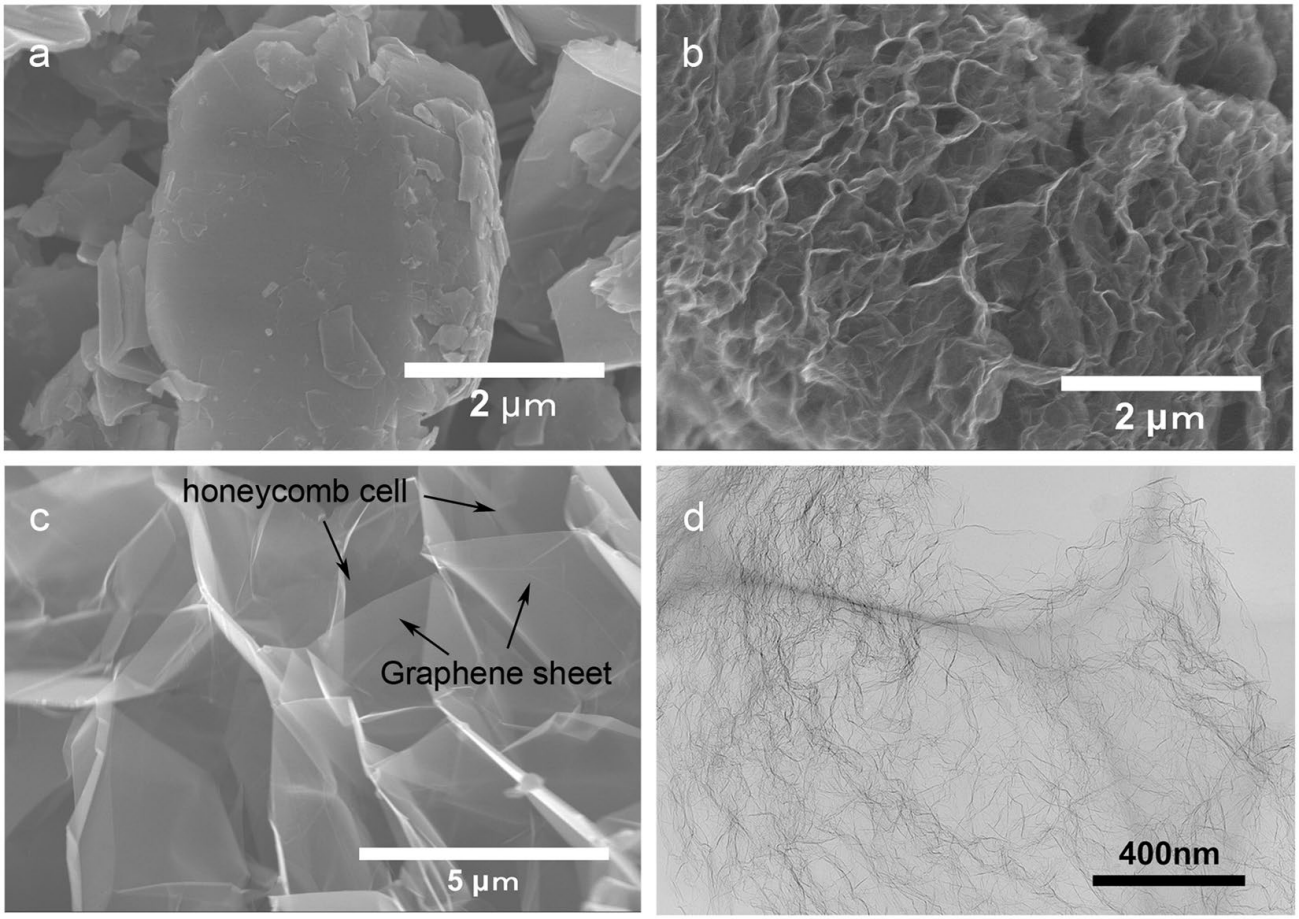
SEM images of (**a**) graphite raw materials for MG, (**b**) PreMG, and (**c**) MG and (**d**) TEM image of MG.

Figure 2 shows the morphologies of Magic G and its precursor materials. Flake natural graphite was used as the raw materials, as shown in Fig. 2(a). These materials were oxidized by a modified Hummer’s method to graphite oxide (GO) and then thermal shocked in N_2_ to 400 °C for 10 min and mild oxidized in air for 30 min at 500 °C to Magic G precursor (PreMG), as shown in Fig. 2(b). Finally, the PreMG was heat treated at 1000 °C for 4 h in N_2_ to obtain MG (Fig. 2(c)). We found that the MG has a honeycomb-like structure with lots of empty cells, with the walls of the honeycomb cell constructed of graphene sheets. Figure 2(d) is a TEM image of MG in which plicate graphene sheets are revealed.

**Figure 3:**
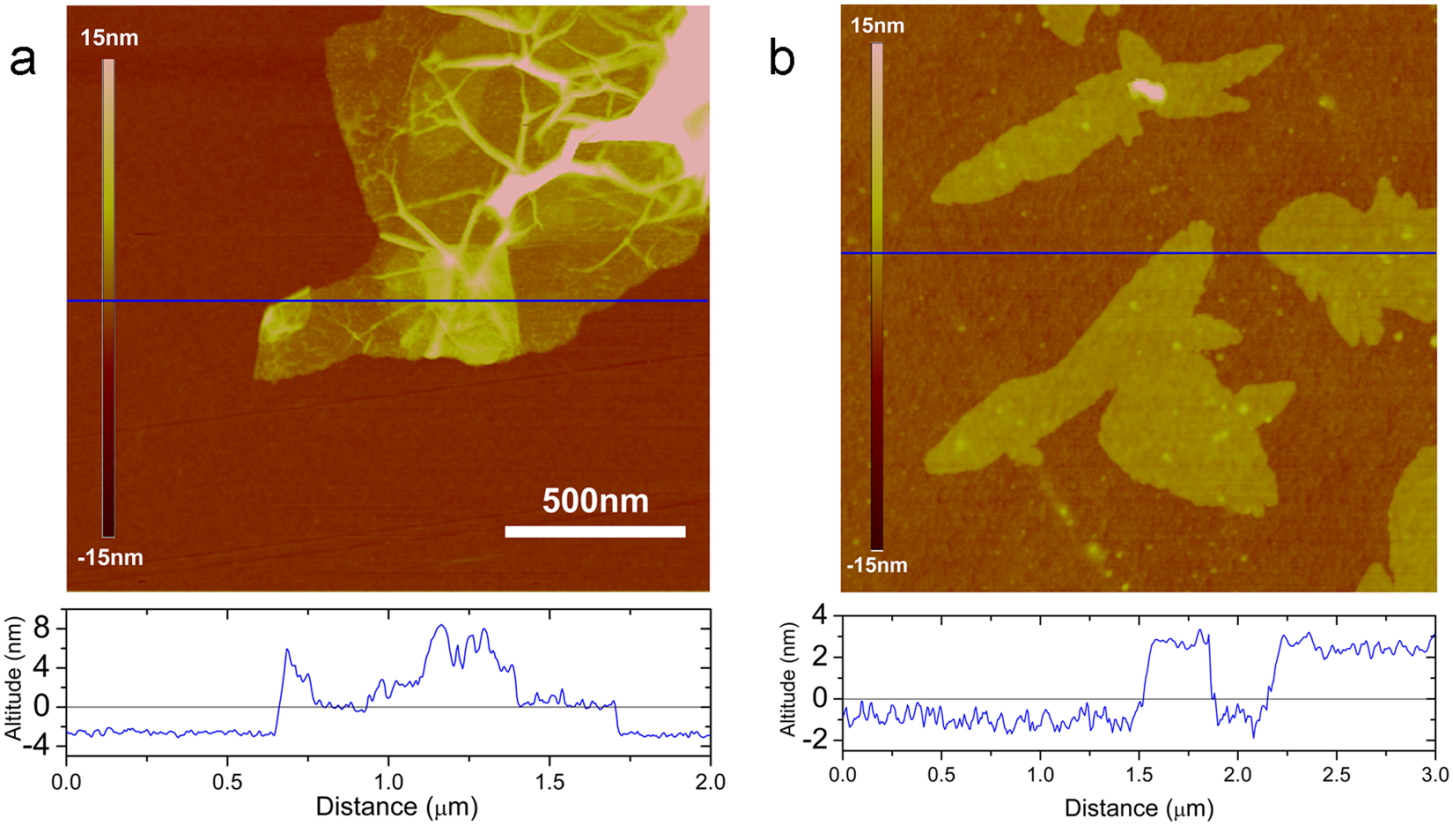
Representative AFM images and the cross sectional high profiles of (**a**) PreMG and (**b**) MG.

Atomic force microscopy (AFM) was also used to characterize PreMG and MG. PreMG showed a wrinkled morphology, which is consistent with the SEM image in Fig. 2(b). The folded morphology came about due to the large quantity of functional groups after oxidation in air (Fig. 3(a)). The MG showed a relatively flatter morphology than PreMG, as shown in Fig. 3(b). We can also see from Fig. 2(c) that the walls of the honeycomb cell graphene sheets are much flatter than those of PreMG. Thickness profiles of the line analysis, shown at the bottom of each image, indicate that the thickness of the MG is about 3 nm.

**Figure 4:**
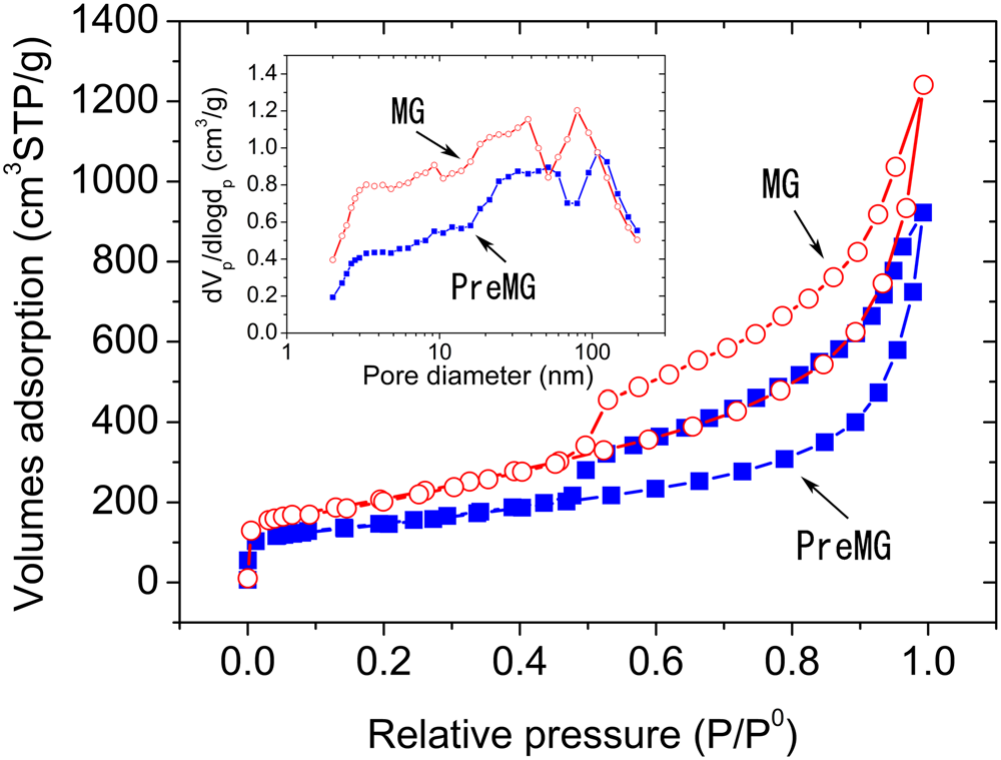
Nitrogen absorption isotherm of PreMG and MG. The inset graph is the pore size distribution of PreMG and MG.

Nitrogen adsorption and desorption isotherms and pore distribution of PreMG and MG are shown in Fig. 4. The multipoint Brunauer-Emmett-Teller (BET) specific surface areas of PreMG and MG are 505 m^2^/g and 1051 m^2^/g, respectively. The greatly increased surface area is attributed to the development of expanded graphene sheets and increased defects by the decomposition of functional groups that were heated at 1000 °C. The pore distribution of PreMG and MG were analyzed by both MP method (0.4–2 nm) and BJH method (2–200 nm). However, pores smaller than 2 nm were not detected by the MP method, which indicates that there were no micropores for either PreMG or MG. The pore volume increased from 1.3 cm^3^/g to 3.6 cm^3^/g while the average pore size increased just slightly, from 12.8 nm to 13.8 nm, from PreMG to MG. The increase of pore volume is due to the evolution of the honeycomb structure from PreMG to MG, which is consistent with Fig. 2(b,c). The high surface area of the MG could benefit to a uniform coating on the surface of active materials to reduce the resistance and the large pore volume of MG may be useful to absorb more electrolytes for charging and discharging.

**Figure 5:**
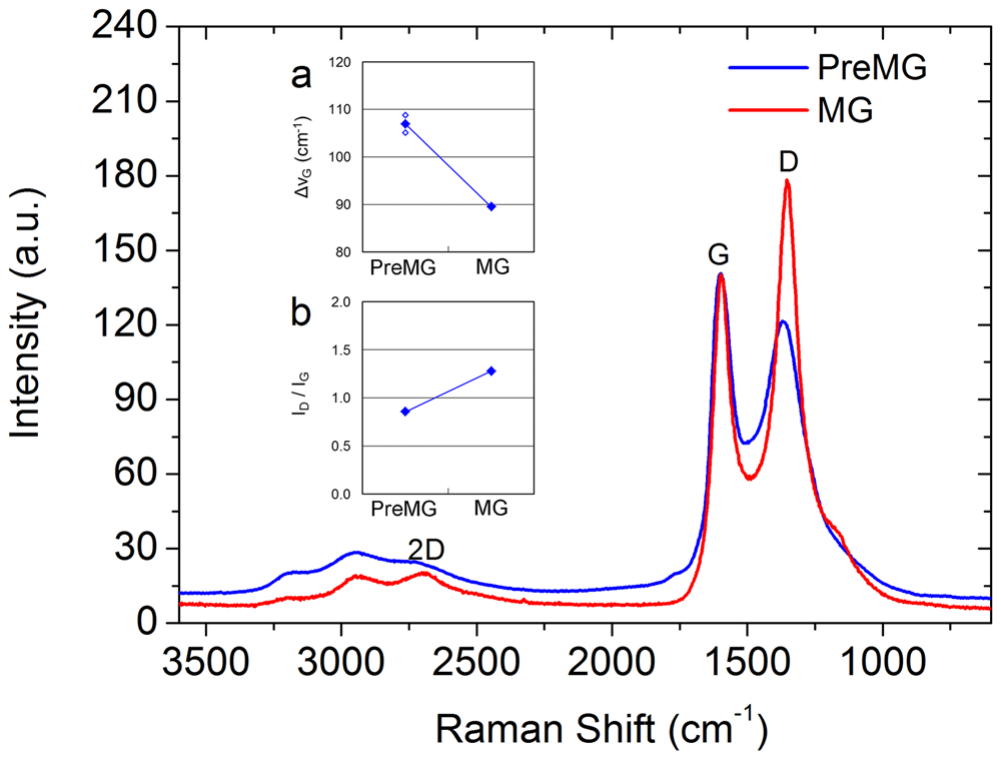
Raman spectroscopy of PreMG and MG. The inset compares (**a**) ΔνG, full width half maximum of G peak with (**b**) I_D_/I_G_, edge ratio of carbon material.

Raman spectra were used to characterize the structure of PreMG and MG carbon materials with normalized G peak intensity. Both PreMG and MG were detected as low crystallized carbon, which suggests a short π electron conjugation length^22–24^. ΔνG corresponding to the stretching vibration mode of graphite near 1580 cm^−1^ is shown in Fig. 5(a). The sharper peak (less ΔνG) means a higher graphitization degree of MG, which indicates that MG has a higher graphitization degree than PreMG. In terms of I_D_/I_G_, which refer to the ratio of edge parts of carbon materials, the MG showed a higher I_D_/I_G_ than PreMG, which we can attribute to the increased edge defects on the basal plane after heat treatment (Fig. 5(b))^25^. These Raman spectra suggest that MG has a better crystallization than PreMG after heat treatment, which is expected to have high conductivity. However, MG is confirmed as a defect rich carbon material, which may affect the initial coulombic efficiency of the electrochemical properties when adding it to the anode.

**Figure 6:**
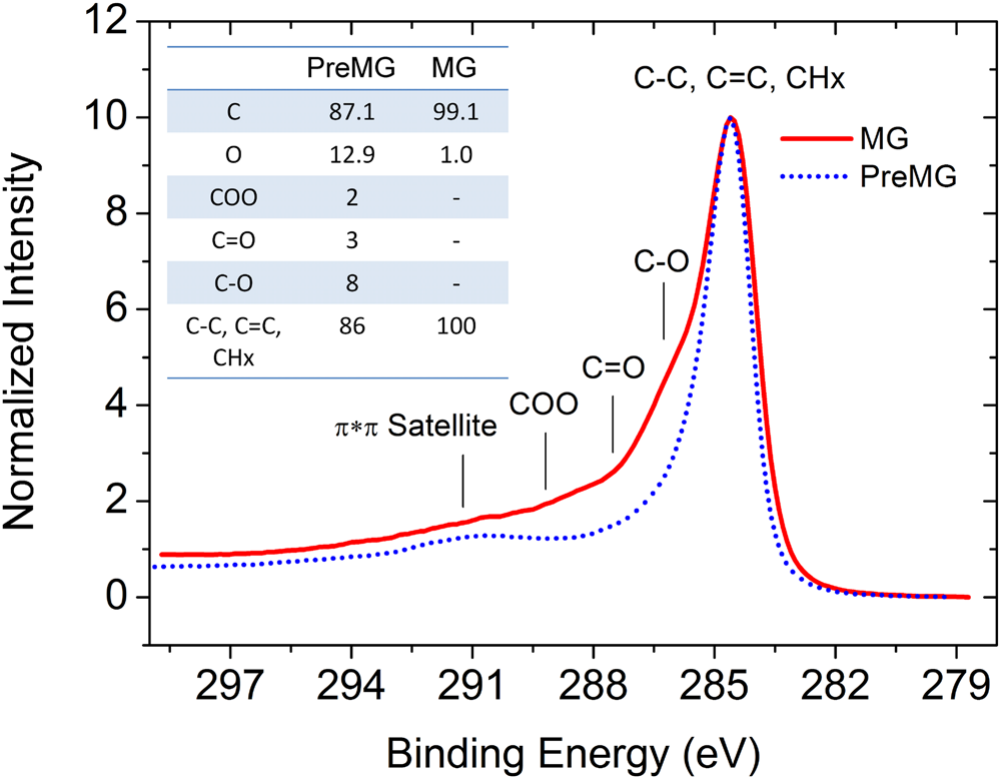
XPS characterization of PreMG and MG. The inset graph is the quantitative analysis of elemental and functional groups at the surface.

The X-ray photoelectron spectroscopy (XPS) analysis is shown in Fig. 6. Both of the samples showed an asymmetric shape with tails on the high binding energy site, which is a shape unique to conjugated systems like graphite^26, 27^. Although C-O, C=O, and O-C-OH were detected in PreMG, these oxygen containing bonding had almost disappeared after the heat treatment and the oxygen content decreased from 12.9 wt% to 1.0 wt%. The functional groups will have an irreversible reaction with lithium ion at initial charge. The low oxygen content of MG seems to have enhanced the conductivity and coulombic efficiency.

**Figure 7:**
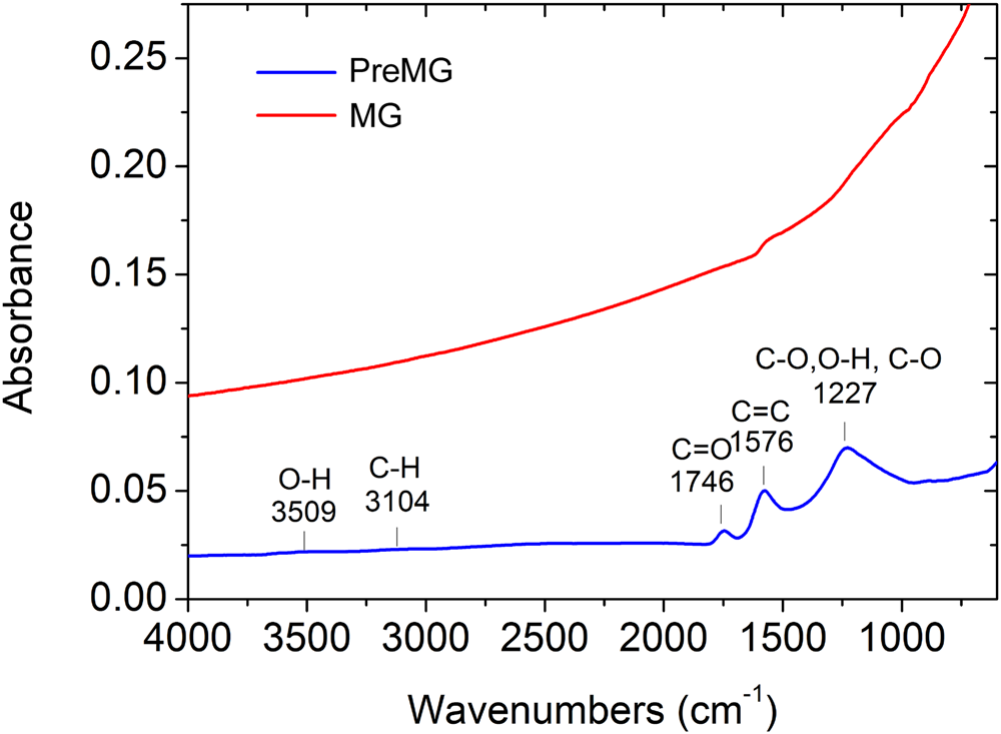
ATR-FTIR characterization of PreMG and MG.

Attenuated total reflectance Fourier transform infrared spectroscopy (ATR-FTIR) was also carried out for the characterization of the functional groups of PreMG and MG, which is showed in Fig. 7. C=O (1746 cm^−1^) and O-H (1227 cm^−1^), which were detected in PreMG, were almost non-existent in MG^28, 29^. Lactone with the assignment region from 1160–1370 cm^−1^ may also be included in the board peak from 1000–1400 cm^−1^ ^30^. C=C bond was detected around 1580 cm^−1^ in MG. The MG spectroscopy showed a higher baseline in lower wave numbers due to the absorption of electrons by conductive materials, indicating the increased conductivity of MG. However, the unique bending vibration at 868 cm^−1^ of graphite was not detected in either the PreMG or the MG, which indicates the amorphous nature of the two.

**Figure 8:**
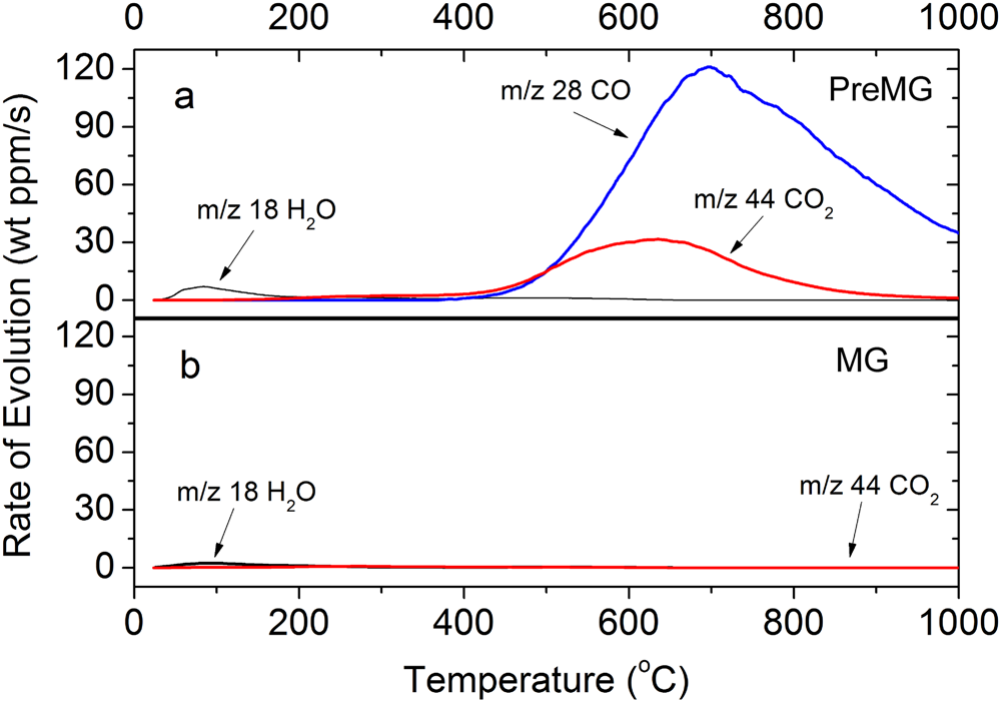
TPD-MS analysis of (**a**) PreMG and (**b**) MG.

Temperature programmed desorption mass spectrometry (TPD-MS) was used to analyze the gas desorption while heating to 1000 °C in He atmosphere (Fig. 8). Peaks of H_2_O (18), CO (28), and CO_2_ (44) were detected in PreMG as the weight ratio of 0.66 wt%, 23 wt%, and 5.3 wt%, respectively. However, only a little bit of H_2_O (0.24 wt) and CO_2_ (0.11 wt%) adsorption was observed in the MG sample^31^. The H_2_O generated at 50 °C is the adsorption water. The CO generation starting from 500 °C is attributed to carbonyl, phenol, or ether functional groups, and CO_2_ may relate to lactone^31–33^. After heat treatment of PreMG, only a little bit of adsorption H_2_O and CO_2_ were detected in MG, which indicates that most of the oxygen containing functional groups had decomposed. The oxygen content of the bulk materials as calculated by TPD-MS was 17.6 wt% and 0.74 wt% for PreMG and MG, respectively. The PreMG had a higher oxygen content than the surface as a bulk material, as learned from the XPS and TPD-MS. However, MG had a higher oxygen content on its surface than the bulk, which may be due to the slight surface oxidization of MG.

**Figure 9:**
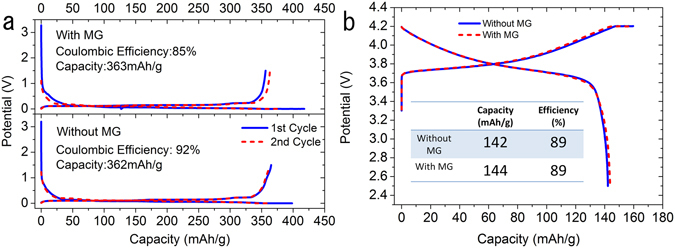
Half-cell initial charge and discharge curves of the reference cell and cell with MG: (**a**) graphite anode vs. lithium metal (**b**) NCM111 cathode vs. lithium metal.

The half-cell initial charge and discharge curves for both anode and cathode with and without 0.5 wt% MG additives are shown in Fig. 9(a,b), respectively. As for the anode materials in Fig. 9(a), the adding of MG did not change the capacity. Both of the anode materials with or without MG showed almost the same capacity, around 363 mAh/g, which is near the theoretical capacity of graphite (372 mAh/g). However, the anode material with MG additives showed a worse coulombic efficiency of 85% compared with the pristine graphite of 92%. This is because MG has large quantities of surface defect that have irreversible reactions with electrolytes to form more SEI on the surface at first cycle, which leads to inferior coulombic efficiency. Figure 9(b) shows the half-cell initial charge and discharge of the NCM111 cathode with and without MG. Similar to the anode materials, the adding of MG did not affect the capacity, which was around 142 mAh/g, but the MG did not give a worse effect to the cathode coulombic efficiency.

**Figure 10:**
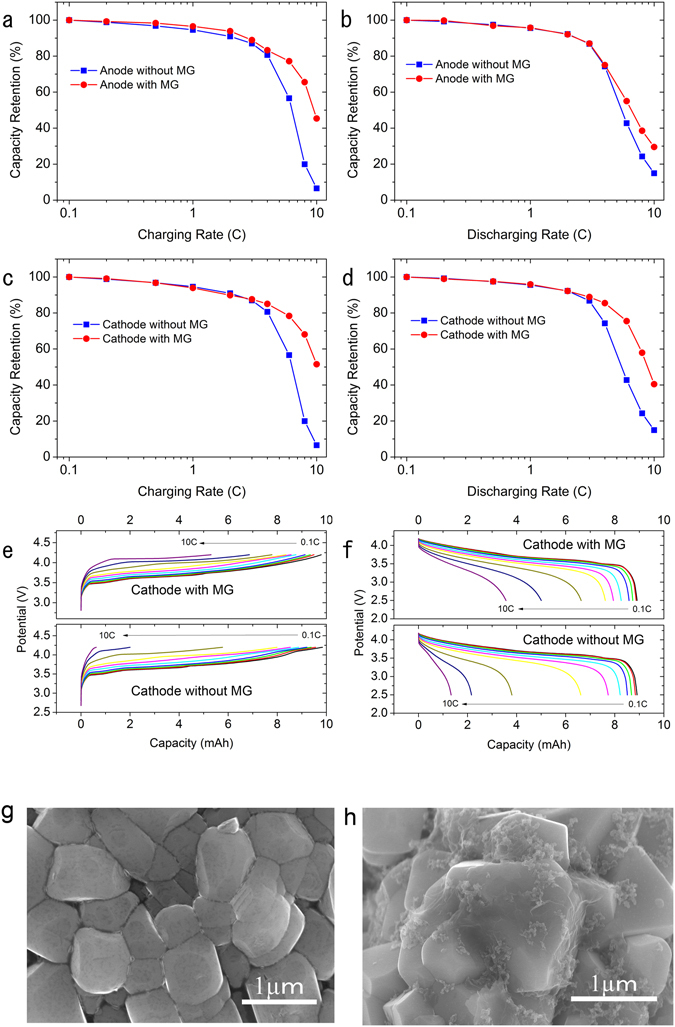
Full-cell charge and discharge rate capability: (**a**) charge rate capability of graphite anode with or without MG (**b**) discharge rate capability of graphite anode with or without MG (**c**) charge rate capability of NCM111 cathode with or without MG (**d**) discharge rate capability of NCM111 cathode with or without MG (**e**) charge curves of 0.1C, 0.2C, 0.5C, 1C, 2C, 3C, 4C, 6C, 8C, 10C with or without MG in cathode, and (**f**) discharge curves of 0.1C, 0.2C, 0.5C, 1C, 2C, 3C, 4C, 6C, 8C, 10C with or without MG in cathode. (**g**) SEM image of cathode electrode surface without MG adding and (**h**) SEM image of cathode electrode surface with MG. Carbon black attached MG were covered on the surface of cathode materials.

The full-cell charge and discharge rate capability of the reference cell and the cell with 0.5 wt% MG added in the graphite anode are shown in Fig. 10(a,b). In Fig. 10(a), the cells were charged at 0.1C, 0.2C, 0.5C, 1C, 2C, 3C, 4C, 6C, 8C, 10C, and discharged at 0.1C from 2.5 V to 4.2 V to evaluate the charge rate capability. The capacity retention at every C-rate is plotted. The rate performance showed improvement from 2C. Specifically, the capacity retention increased from 56% to 77% at 6C (10 min charging) and from 7% to 45% at 10C (6 min charging). This great improvement in charge rate capability, especially at high rates, may be attributed to 1) high conductivity of the MG additives, 2) the honeycomb-like structure with high surface area for better electrolyte adsorption, and 3) MG deposited on the surface of active materials for a smaller charge transfer resistance. In Fig. 10(b), the cells were charged at 0.1C and discharged at 0.1C, 0.2C, 0.5C, 1C, 2C, 3C, 4C, 6C, 8C, 10C from 2.5 V to 4.2 V to evaluate the discharge rate capability. Similarly, the capacity retention increased from 43% to 55% at 6C and from 16% to 30% at 10C, which indicates that adding MG causes faster lithium deintercalation kinetics. As it was showed in Fig. 9(a), the adding of high specific surface area MG to anode could result in lower initial coulombic efficiency. Therefore, the rate capability of cathode with or without MG were also investigated. Figure 10(c,d) show the charge and discharge rate capability of the reference cell and the cell with 0.5 wt% MG added in the NMC111 cathode. Figure 10(c) shows the charge characterization tested under exactly the same conditions as Fig. 10(a), namely, charged at 0.1C, 0.2C, 0.5C, 1C, 2C, 3C, 4C, 6C, 8C, 10C, and discharged at 0.1C from 2.5 V to 4.2 V. The charge curves were plotted in Fig. 10(e), it can be learned the cathode with MG showed higher capacity in constant current (CC) charge, especially in high rate. After adding MG to the cathode, the capacity retention improved from 56% to 78% at 6C, while the 10C rate improved from 7% to 52%. Adding MG to the cathode material is also effective in terms of increasing the charge rate capability, since better electron and ion conductivity of cathode materials resulted from adding MG and from electrolyte supply from MG absorption. As for the discharge rate capability shown in Fig. 10(d), the 6C rate improved from 43% to 76% and the 10C rate improved from 16% to 40%. The discharge curves of each C-rate were showed in Fig. 10(f) that the cell with MG showed larger discharge capacity in high rate. It is clear that adding MG in the cathode has a positive effect on the discharge rate capability of the cell. This is because lithium ions need to intercalate to the cathode active materials (lithiation) while discharging, while the semi conductive cathode materials usually have low electric conductivity as bulk and high charge transfer resistance, which can be improved by adding MG. Figure 10(g) is the SEM image of cathode electrode surface without MG. It can be learned there is almost no carbon black particles attached on the surface of cathode materials although 3 wt% of carbon black was added. This is because the carbon black is more likely to accumulate to the spaces among active material particles instead of attached on their surface. However, it is clearly showed in Fig. 10(h) that the cathode surface was particle covered by MG and the carbon black particles were attached on the surface of MG, which could provide a lower surface resistance. Finally, based on the electrochemical properties, we conclude that the MG is effective for improving rate capability for both the anode and cathode of lithium ion batteries.

**Figure 11:**
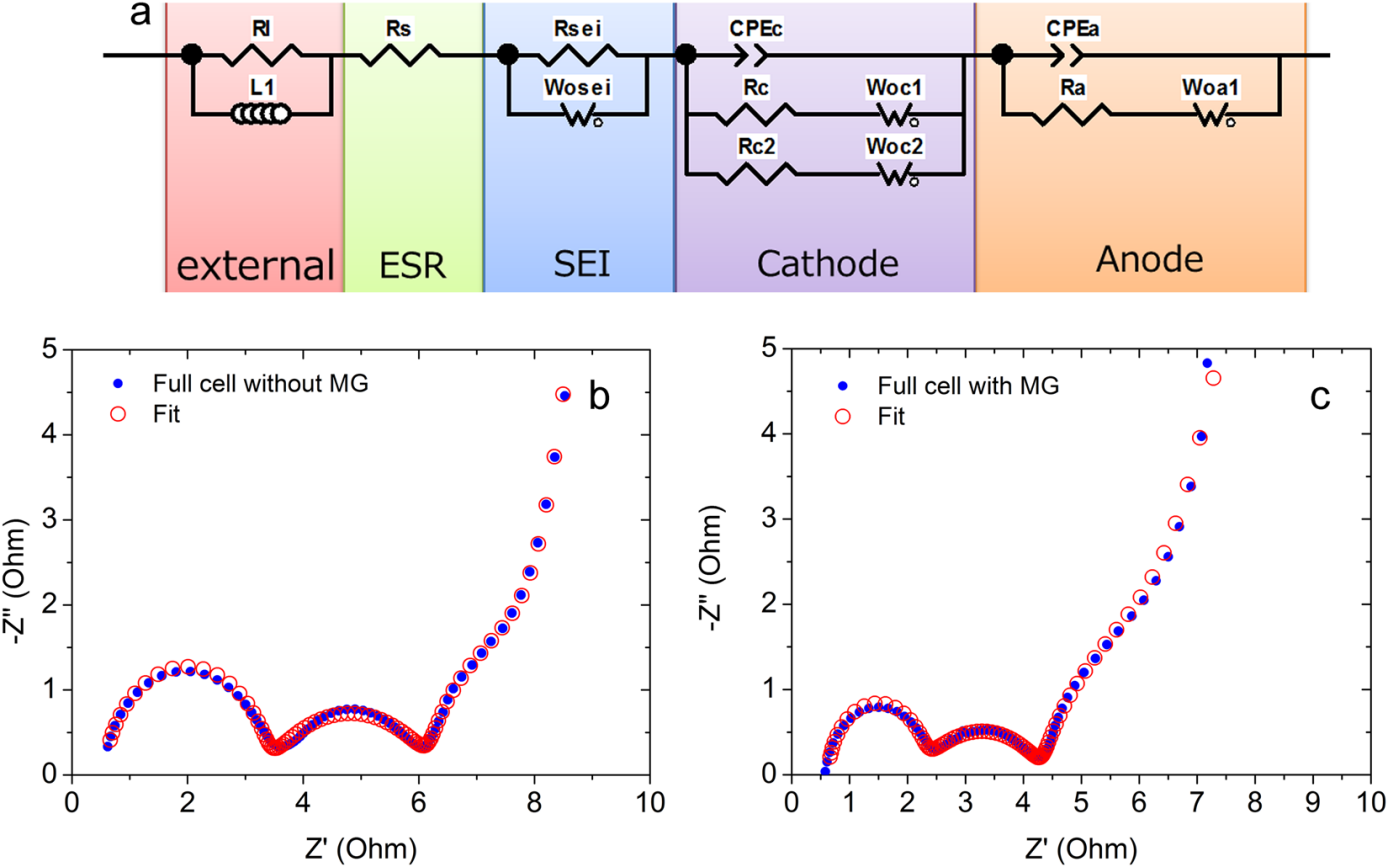
Electrochemical impedance spectroscopy (EIS) analysis: (**a**) equivalent circuit (**b**) EIS curve of cell without MG, and (**c**) EIS curve of cell with MG.

Electrochemical impedance spectroscopy (EIS) was used as the impedance analysis to clarify the impedance response of the cell^34, 35^. The typical Nyquist plot of a LiB cell usually has semicircles in the high and mid-frequency range. The first big semicircle was reported to be mainly attributed to the cathode and the second semicircle to the anode^36, 37^, and Osaka *et al*. reported that the first semicircle belongs to the anode and the second semicircle to the cathode^38–40^. However, after a systematic comparison of a SOC50 anode/anode symmetric cell, SOC50 cathode/cathode symmetric cell, and SOC50 anode/cathode full cell, we conclude that the first semicircle is attributed to the cathode and the second semicircle to the anode in a laminated full cell with graphite as anode and NCM111 as cathode. In addition to the two main semicircles of cathode and anode, a straight line near 45 °C was included and relates to the diffusion process inside of the active material. At ultra-low frequency (below 1 mHz), a nearly vertical line is revealed that corresponds to the pure capacitor behavior. We have proposed the equivalent circuit shown in Fig. 11(a) for this cell on the basis of an equivalent circuit with components and interfaces in lithium ion batteries. The electrochemical reactions of both cathode and anode are expressed with a parallel connection of interfacial capacitance and connected charge transfer resistance with Warburg impedance in series. The circuit also contains an equivalent serial resistance (Rs) and external inductive component consisting of an inductor and resistor (L1 and R1) related to the wiring between the electrodes with the measuring equipment, including the wounded current collector. The cathode part is represented as a model composed of active materials with two different radiuses. Two sets of series connection of diffusion element and charge transfer resistance should be connected in parallel with a capacitance between the electrolyte and the electrical connection between particles. The variation of the capacitance in particles is represented as the constant phase element (CPE). The capacitors for particles with both radiuses should connect in parallel and be simplified as one CPE^35^. In order to consider the component of SEI, lithium ion was assumed to move into SEI by migration. The impedance component representing the SEI was made as a parallel connection of resistance and the capacitance of the SEI layer^34, 38, 40^. Figure 11(b,c) show the typical EIS curves with fitting data of the reference cell and the cell with added MG. The equivalent serial resistances (ESR) of both cells without and with MG are calculated as 0.104 Ω/cm^2^ and 0.094 Ω/cm^2^, respectively. The adding of MG could reduce 9.6% of the bulk resistance. The normalized charge transfer resistance of the cathode (Rc) of both cells was 0.106 Ω/cm^2^ and 0.072 Ω/cm^2^, respectively. The cathode charge transfer resistance was reduced 32% with the MG addition. We can also calculate that the charge transfer resistance of anode (Ra) without and with MG is 0.44 Ω/cm^2^ and 0.4 Ω/cm^2^, respectively. We found that 9% of the anode charge transfer resistance was reduced by the MG adding. Moreover, the diffusion curve shown in Fig. 11(c) has a relatively smaller slope than the one in Fig. 11(b) at low frequency, which indicates a higher electrochemical double-layer capacitance caused by MG.

**Figure 12:**
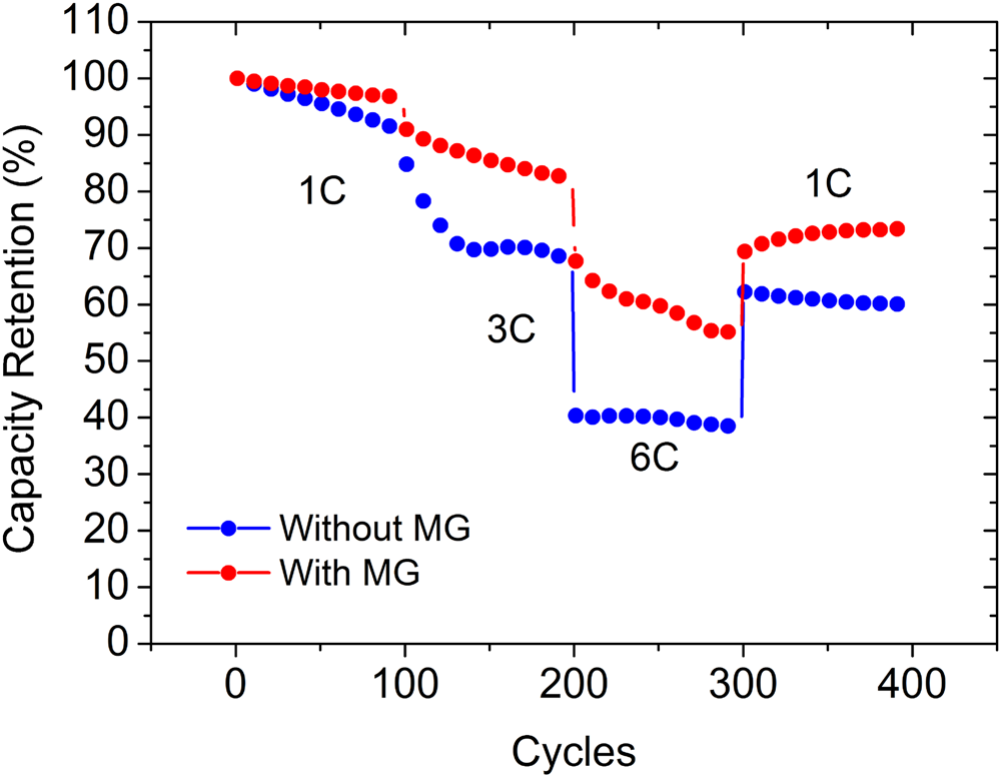
Full cell rate cycling of reference cell and cell with MG at 1C, 3C, 6C.

Figure 12 shows the cyclability of the reference cell and cell with MG additives at 1C, 3C and 6C in full cell. The reference cell and the cell with MG showed capacity retention of 91% and 96%, respectively in the first 100 cycles at 1C. After the following 100 cycles at 3C, the reference cell dropped to 68% compared with cell with MG of 82%. Finally, the capacity retention of the reference cell dropped to 38% after another 100 cycles at 6C, while the cell with MG just dropped to 55%. The greatly improved rate cyclability may be attributed to the high electric conductivity, high ability of electrolyte absorption, and improved surface charge transfer resistance after the MG was added to the cell.

## Conclusion

We have designed and fabricated a honeycomb-like porous graphene sponge additive for both anode and cathode materials to increase the charge and discharge rate capabilities by increasing the electron conductivity, enhancing the adsorption of electrolytes, and reducing the active materials charge transfer resistance. The cell with MG additive showed a great improvement of capacity retention in high rate charging, discharging, and cycling. We consider the porous graphene-based additive a promising additive material for next-generation lithium ion batteries with both high energy density and good rate capabilities for EVs and PHVs. In the future, we will work on optimizing the structure of the additive to further increase the beneficial properties for fast chargeable LiBs.

## Methods

### Synthesis of Magic G

Natural graphite was used as the raw material and the modified Hummer’s method was used to change the graphite into graphite oxide (GO)^41, 42^. First, graphite and NaNO_3_ were mixed together in a flask and then H_2_SO_4_ (100 ml, 95%) was added to the flask, which was kept in an ice bath while being stirred. Potassium permanganate (8 g) was added to the suspension slowly to avoid overheating. The mixture was then stirred at room temperature for 2 h and the color of the suspension became bright brown. Distilled water (90 ml) was added to the flask and then stirred. The temperature of the suspension quickly reached 90 °C and the color changed to yellow. The diluted suspension was then stirred at 98 °C for 12 h. H_2_O_2_ (30 ml of 30%) was added to the mixture. For purification, the mixture was washed by rinsing with 5% HCl followed by deionized water several times. After that, the suspension was centrifuged at 4000 rpm for 6 min. After filtration and drying in a vacuum, the graphene oxide was obtained as black powder. The as-synthesized graphite oxide was then thermal shocked at 400 °C for 20 min in N_2_ atmosphere followed by a mild oxidation in dry air at 500 °C for 30 min to activate the graphene surface as the Magic G precursor (PreMG). In the next step, the PreMG was heated in N_2_ atmosphere to 1000 °C at 5 °C/min and kept at 1000 °C for 6 h for a complete reduction of activated PreMG to MG.

### Characterization

The morphology of the products was carried out by field-emission scanning electron microscopy (FE-SEM) (Hitachi, SU8000, 5 kV) and transmission electron microscopy (TEM) (Hitachi, H-90000UHR, 300 kV). Atomic force microscopy (AFM) (Bruker AXS Nano Scope V Dimension Icon, tapping mode, scan range 2–10 μm, scan speed 0.3–0.5 Hz) was also used for the morphology characterization. The AFM samples were made by dispersing the carbon in a mixture of ethanol and water (1:4) in 0.5 mg/L and dropped on a silicon wafer at RT for 24 h. Fourier transform infrared (FT-IR) spectra of the samples were captured by a FT-IR spectrophotometer (Varian 7000FT-IR, resolution 4 cm^−1^, cumulated number 512) using an attenuated total reflection method (ATR crystal Ge, incident angel 45°). Raman spectroscopy was performed on NRS-7000 series with a maximum resolution of 0.7 cm^−1^/0.3 cm^−1^ and the measurement range of 50 to 8000 cm^−1^. The gas adsorption was measured by BELSORP18PLUSUS-HT with the samples pretreated in 200 °C for 5 h. The specific surface area was calculated by Brunauer–Emmett–Teller (BET) theory and the pore distribution was analyzed by both MP (0.4–2 nm) and BJH (2–200) methods. Temperature programmed desorption–mass spectrometry (TPD-MS, GC/MS QP2010plus10) was used to analyze the mass spectrometry from room temperature to 1000 °C at the speed of 10 °C/min in He atmosphere. The electrochemical properties of the proposed materials were characterized in both half-cell and full-cell configuration. The cut potential range for the half cell anode measurement was from 0 V to 1.5 V while the cathode and full cell charge and discharge was carried out in the voltage range of 2.5 V to 4.2 V.

### Cell fabrication

The synthesized MG was characterized as the additives in anode and cathode for both half-cell and full-cell, respectively. The reference anode electrode was prepared by coating a mixture of granulated natural graphite (CGB-20, Nippon Graphite Industries), carbon black, carbonxymethy cellulose (CMC), and styrene-butadiene rubber (SBR) with the weight ratio of 97%:1%:1%:1% on a copper film with the mass loading of 60 g/m^2^ for a single side. The MG added anode electrode was prepared by coating a mixture of granulated natural graphite, carbon black, MG, CMC, and SBR with the weight ratio of 97%:0.5%:0.5%:1%:1% with the same mass loading. The density of anode electrodes was pressed at 1.49 g/cc. The thickness of the copper foil is 10 μm.

The reference cathode electrode was prepared by coating a mixture of lithium nickel cobalt manganese oxide (NCM111, BASF), carbon black, and polyvinylidene fluoride (PVDF) with the weight ratio of 93%:3%:4% on an aluminum film with mass loading of 127 g/m^2^ for a single side. The MG added cathode electrode was prepared by coating a mixture of NCM111, carbon black, MG, and PVDF with the weight ratio of 93%:2.5%:0.5%:4% on an aluminum film with mass loading of 127 g/m^2^ for a single side. The density of the cathode electrodes was pressed at 2.78 g/cc.

Regarding the half-cell configuration, the anode and cathode were assembled with lithium metal deposited copper film as a laminate cell. The full cell was assembled with the as-prepared anode and cathode with the A/C ratio of 1.2. 1 M LiPF_6_ EC/DEC (3:7) with was used as the electrolyte. An 8 Ah laminate cell was fabricated with the same anode and cathode as described above (30 layers of cathode and 31 layers of anode) and the energy density was confirmed as 162 Wh/kg. However, all the rate capability and cycling performance was tested in a small type laminate cell (anode: 23 mm × 24 mm, cathode: 22 mm × 23 mm) with the same anode and cathode electrodes.
